# The Work of the Imperial Cancer Research Fund

**Published:** 1905-09-23

**Authors:** 


### HOSPITAL CLINICS.

### THE WORK OF THE IMPERIAL CANCER RESEARCH FUND.

(*Concluded from page 429.*)

THE final section of this volume [1] is devoted to a consideration of the chief of the many hypotheses, which have from time to time been called into service for the explanation of the phenomena of malignancy in new growths. Some of these have already passed out of currency, and some are still with us, but the section is important as postulating, in the light of recent researches, certain established characteristics of malignant new growths, all of which must be accounted for before any theory can be considered acceptable. These postulates are as follows:—

1. That the primary site of a cancerous growth is a circumscribed area.
2. That a cancer is relatively independent of its surroundings.
3. That cancer-cells are capable of continuous growth.
4. That infiltration and destruction are characteristic of cancer.
5. That cancers are capable of producing metastatic deposits.
6. That cancer appears with increasing frequency as age advances.
7. That cancer-cells show a single differentiation wherever they appear. (That is, that the type of cell for any given tumour is always the same.)
8. That cancer is, with limitations, capable of transmission.
9. That cancers are unaccompanied by specific symptoms.

A thoughtful consideration of these postulates will assist the realisation of the important work accomplished by the Imperial research authorities. We have suffered too much in the past from the random assumption of hypotheses without a sufficiently systematic investigation of the facts, which any acceptable hypothesis must be called upon to explain. It would appear that now at last we have some definite data to guide the imaginative amongst us.

The hypothesis of Thiersch and Waldeyer was to the effect that under normal conditions an equilibrium existed between the connective and epithelial tissues, and that the disproportionately rapid aging of the former destroyed that equilibrium with the result that proliferative powers inherent in the epithelial tissues were no longer held in check. This hypothesis fails to explain the circumscribed nature of the primary site, the infiltrative and destructive characters, the formation of metastases and the power of transmission possessed by new growths.

Cohnheim secured a wide currency for views which ascribed the origin of cancers to "embryonic rests," that is to say, groups of cells which by some developmental error had been cut off from the rest of the organism, and while undeveloped yet retained potential powers of growth. These remains of embryonic tissue were assumed to renew their proliferative powers under the influence of various agencies. This hypothesis disregards the fact that such embryonic rests, once they resumed their proliferative phase, might be expected to proceed along the normal lines of differentiation common to embryonic cells. Did they do so the hypothesis must fail, for the growth would be no cancer, as we know it, a mass of cells of uniform type. In addition Cohnheim postulates infiltration, destruction, and metastasis formation without explaining them, and regards the peculiar age-incidence of cancer as of no importance. Lastly, the established transmissibility of cancer leaves the theory quite untenable.

The strong points of these two hypotheses have been combined by Ribbert. He has attempted to prove that the dislocation of epithelium from its normal surroundings elicits latent powers of unlimited proliferation. Where the cells of a cancer are altogether undifferentiated and embryonic, the theory of Cohnheim is held to cover the ground; where they are differentiated (though the type in any given tumour remains of course the same), Ribbert assumes a "post-natal rest," that is an isolation of differentiated cells by some process of chronic inflammation. But there is no evidence of latent powers of growth either in embryonic or in adult epithelium in any way comparable with those revealed by the successful propagation of cancers. The growth which follows successful tissue-grafting is limited in all cases, while in the case of cancer it may be unrestricted. Moreover the hypothesis practically fails in the case of sarcomata.

A fourth hypothesis ascribes the process to a parasite. The researches of the Fund have supplied the following evidences against this theory.

(*a*) The disease is common to all vertebrates, and produces identical histological lesions in all species subject to it, yet cannot be transmitted from one species to another.

(*b*) Infective diseases are known which appear to attack one species only, for instance syphilis; but then the lesions of syphilis are not found in any other species. On the other hand, infective diseases of wide distribution such as tuberculosis, though sometimes conveyed with difficulty from one species to another, yet are transmitted with ease to animals of the same species. This is not the case with cancer, with the solitary exception of the mouse.

(*c*) All known infective diseases call forth a reaction on the part of the infected host. This is not the case with cancer.

(*d*) In the experimental propagation of cancer the elements of the soil are insignificant in importance, and merely subserve the needs of the tumour-cells introduced; in tumours resulting from infection, for instance, infective granulomata such as the venereal disease of the dog previously alluded to, the introduced cells are merely vehicles, and die, while the whole tumour, apart from the pathogenic micro-organisms, is supplied by the reaction of the soil.

(*e*) No sporadic tumours have been observed in

[1] Scientific reports of the Imperial Cancer Research Fund 1905. Part 2. London. 2s. 6d.

animals living in close contact with other cancerous animals of the same species.

Lastly comes the hypothesis originated by Beatson and elaborated by Professor Farmer and others, that malignant new growths are allied to reproductive tissue. The processes of cell-division occurring in reproductive organs are essentiallv different from those observed in the somatic cells of the body. Without going into full-detail it may be mentioned that the difference lies in the character of the nuclear figures which precede cell-division. The upholders of this theory assert that the reproductive type of cell-division is constant in malignant tumours, and feel justified in deliberately correlating the appearance of malignant neoplasms with the result of a stimulus which has changed the normal somatic course of cell-development into that characteristic of reproductive tissue. The hypothesis comes to this, that under the influence of some stimulus, such for instance as chronic irritation, differentiated somatic cells become transformed into reproductive cells. An integral part of the explanation is therefore based upon the view that the reproductive tissues proper arise merely from a transformation of somatic tissues, and are not inherently different from them. The criticisms of this theory offered by the Fund report are very meagre. It does not explain the phenomenon of single differentiation, nor the peculiar age-incidence which forms so remarkable a feature of malignant disease.

It will be seen from the abstracts of the report supplied in these columns that the work of the Fund has been largely negative: that is to say that many misconceptions have been brushed aside by investigation. The cause of cancer remains still obscured, but much solid progress has been made in our knowledge of the processes of growth implied by it. The first step in advance is the rejection of traditional errors, and in this direction so much has been accomplished that we may look forward with hope to subsequent years of more positive progress.

